# Scientific Items

**Published:** 1878-04-20

**Authors:** 


### *SCIENTIFIC ITEMS.*

### THE ELECTRIC LIGHT IN PARIS.

A lecture on the electric light was lately given by M. Jamin in a large amphitheatre of the Sorbonne in Paris, and the room was crowded with an audience of two thousand persons. Some accident occurred to the gas-metre, so that the gas was suddenly extinguished, and the audience would have had to be sent away but for the subject of the lecture, which gave a capital occasion for practical demonstration of the lecturer's statement that electric lighting had now entered on the phase of practical exploitation. The regulators, which were meant only for experimental demonstration, served to illuminate the hall beautifully throughout the evening. The arc appeared in fourteen lamps, each with the brightness of a hundred Carcel burners, being fed by electro-magnetic engines of various systems, driven by two steam engines. The fourteen jets could all be extinguished or re-lit simultaneously for projection on the screen, and the process was in all respects successful. M. Jamin was assisted by the well-known inventors, MM. Lontin, Gramme and Jablochkoff.—*Boston Jour. Chem.*

### DISGUST PHILOSOPHICALLY CONSIDERED.

An interesting paper on "The Causes of Disgust," by M. Charles Richet, is printed in a late number of the *Revue des Deux Mondes.* The author considers disgust, when analyzed philosophically, to be an instinctive sentiment of protection, varying with species and with alimentation, habits, and education of individuals. But under this apparent diversity there is the general law of finality; and it is not by chance that our disgust attaches to such and such a being or substance, but in consequence of the hereditary instinct which has apprised our ancestors that these animals and substances might be dangerous for us. Disgust sometimes attaches to the total form of objects, and may diminish and become extinct as scientific analysis disjoins the parts of the repugnant whole. Thus, a spider, viewed as a whole, is a repulsive creature; but take a leg or an eye of it and study in the microscope the marvellous arrangement of these organs, and the sight will awaken admiration instead of disgust. Again, habit is evidently an important factor in the feelings of disgust. Thus, to eat frogs or snails is repugnant to us, yet we eat without disgust such things as black pudding, tripe, liver, high game, and decayed cheese. The aversion to horse-flesh is not readily accounted for, except by habit.

### TELEGRAPHING WITHOUT WIRES.

Professor Loomis' theory of telegraphing without wfres is based on a current of electricity which he has demonstrated exists at different heights, and which transmits communication between two perpendicular wires reaching it, whatever the distance may be. In his experiments he has employed kites, substituting copper wire for the string. By using the Morse apparatus, signals were transmitted between kites eleven miles apart. It has been suggested that as ærial currents are found at high altitudes, it may be possible to send messages across the ocean through the sky.—*Leslie's Popular Monthly.*

### PERSISTENCE OF THE RETINAL IMAGE.

Dr. Gorini makes allusion to supposed persistence upon the retina of images perceived during the last moments of life. Once when reading he fell asleep, for, he believes, an hour. On awaking he saw the wall, which was opposite to him, lit up by his lamp and covered with typeforms of great size, forming words and lines exactly as was the book which he was reading when he fell asleep.

